# Comparison of multiple flatfoot indicators in 5–8-year-old children

**DOI:** 10.1515/med-2021-0227

**Published:** 2021-02-01

**Authors:** Saidas Žukauskas, Vidmantas Barauskas, Emilis Čekanauskas

**Affiliations:** Department of Paediatric Surgery, Lithuanian University of Health Sciences, Pramonės pr. 47-24, Kaunas, Kauno m., LT-50461, Lithuania; Department of Paediatric Surgery, Lithuanian University of Health Sciences, A. Mickevičiaus g. 9, LT-44307, Kaunas, Lithuania; Department of Paediatric Surgery Pediatric Orthopedics – Traumatology Unit, Lithuanian University of Health Sciences, A. Mickevičiaus g. 9, LT-44307, Kaunas, Lithuania

**Keywords:** foot posture index, foot assessment, pediatric flatfoot, foot prints, Chippaux–Smirak index

## Abstract

**Background:**

The foot posture is age dependent. The purpose of this study was to investigate the relationship between the 6-item version of the foot posture index (FPI) and other clinical, foot anthropometric, radiological measurements for the foot position in 5–8-year-old children.

**Methods:**

A total of 301 participants with a mean age of 6.4  ±  1.14 years were enrolled in the study. Children were examined physically, clinically, and radiologically to measure the FPI and navicular drop (ND) test, resting calcaneal stance position (RCSP) angle, Chippaux–Smirak index (CSI), Staheli index (SI), calcaneal pitch (CP) angle, talocalcaneal angle (TCA), and the first lateral metatarsal angle. Tibial torsions, internal rotation of the hip as an indirect method of femoral anteversion, and Beighton scale were analyzed for factors associated with flatfoot prevalence.

**Results:**

The study included children with normal and flexible flatfeet. Statistical analysis showed a significant FPI score correlation with other parameters (SI, CSI, RCSP, ND, CP, TMA, and TCA showed strong and moderate correlations, *p* < 0.001). Overall, the strongest associates are CSI (*β* = 0.34) and ND (*β* = 0.28). Other indicators have relatively small relationships with the FPI.

**Conclusion:**

A positive correlation was observed between FPI-6 and ND test, CSI in 5–8-year-old children. All three prominent foot posture indicators (FPI-6, ND, and CSI) might be used as a primary or preferred tool in clinical practice.

## 1 Introduction

Flatfoot is a common foot posture in children and a frequent concern for parents by the appearance of children’s feet [1,2]. The foot posture is age dependent and the exact time when the medial longitudinal arch of the foot is formed is still not exactly known [2]. According to studies, a flat arch is typical for children at birth but resolves spontaneously until 6 years of age [1,3]. In contrast to other studies, the arch of the child’s feet is fully formed in about 10 years of age [2,4].

In most clinical practices, a non-dynamic assessment of children’s feet is performed to classify foot posture excluding pathological feet [1,5]. This includes four main methods of assessment: (a) non-quantitative visual assessment, (b) anthropometric measurements (resting calcaneal stance position [RCSP] angle, navicular drop [ND] test, and medial longitudinal arch angle), (c) various footprint-based analysis (Chippaux–Smirak index [CSI], Staheli arch index [SI], etc.), and (d) radiological examinations with various measurements. This includes MRI, ultrasound, and laser scanners [5,6,7,8]. Radiographic procedure is the golden standard for evaluating the medial longitudinal arch height [9,10], even though radiographic measures typically provide only a uni-plantar assessment of the foot posture [5]. In contrast, the foot posture index (FPI-6) is a clinical and multiplanar tool that displays three planes for the assessment of the foot. FPI-6 classifies the posture of the foot in pronated, supinated, or neutral positions [5,10]. Previous studies showed that FPI-6 is a reliable test for measuring the foot position; therefore, it has acquired popularity over the years [1,4,5,11,12].

Another biomechanical parameter is widely used to diagnose resting foot posture – RCSP [1,5,13]. The RCSP has a high degree of reliability, independent of age, height, and weight [13]. According to studies, validity and reliability of RCSP angle are relatively less common than those of FPI-6 [5], especially at different ages of the child.

ND represents the medial ‘drift’ of the navicular bone from neutral to resting stance position. It reflects movements of the medial longitudinal arch and is associated with the pronated foot [6]. In a cohort of adults, the FPI-6 showed an excellent correlation with the ND [10]. However, a recent study has concluded an ND as an unreliable measure with only fair agreement across test sessions [5].

Various footprint-based analyses for foot arch assessment have been developed and are used widely. The CSI and SI are regarded as reliable by many investigators in calculating arch development and are recommended in screening for flatfoot in preschool-aged children [7,14,15]. The FPI-6 showed a good correlation with the CSI and SI in adults [10].

The configuration of the arch is determined by age, height, weight, foot progression angle, sex, joint hypermobility, tibial torsion, femoral anteversion, hindfoot alignment, and occurrence of physiological knock knee [16].

Because of this, in our study, inclusion criteria were set for 5–8 years children after the rapid progression of the plantar arch. In addition, the relationship between FPI-6 and RCSP, ND, SI, CSI, X-ray measurements – calcaneal pitch (CP) angle, talocalcaneal angle (TCA), lateral first metatarsal angle (TMA), tibial torsions, and internal/external rotation of hip is currently unclear. Therefore, the purpose of this study was to investigate the relationship between FPI-6 and other clinical (tibial torsion, femoral anteversion), footprint (CSI, SI), foot anthropometric (ND, RCSP), radiological (TCA, CP, and TMA) measurements for foot position in 5–8-year-old children.

Results of this study could provide information on whether ND, RCSP, SI, CSI, X-ray measurements, and clinical measurements could be used as a clinical, radiological measuring tool for pediatric flatfoot. This is the first study to reveal a correlation between FPI-6 scores for foot posture and footprint parameters (CSI, SI), clinical parameters (tibial, femoral torsions, RCSP, and ND), and radiological parameters (TMA, CP, and TCA) in 5–8 years flexible flatfeet and non-flatfeet children.

## 2 Methods

### 2.1 Patients

The sample of interest was 5–8-year-old children. Between April 2019 and February 2020, a total of 301 participants (146 boys and 145 girls) with a mean age of 6.4  ±  1.14 years were enrolled in the cross-sectional comparative study. The study participants visited the hospital for general orthopedic and foot examination without pain as a regular checkup that is mandatory in the country. The eligibility was defined as follows:

- Inclusion criteria: asymptomatic flexible flatfeet (positive Jack’s test, FPI ≥ 6); non-flatfeet, with no evident joint deformities (FPI 0–5); aged 5–8 years;
- Exclusion criteria: foot pain, injury to the lower limbs during the previous 12 months, congenital abnormalities, cerebral palsy, motor dysfunction, prior foot surgery, the use of foot orthoses, a fixed foot deformity.

We obtained information about age, sex, body weight, height, and underlying diseases. The sample was well balanced regarding gender and age, with a slight overrepresentation of a 5-year-old group. Table 1 shows the demographic and anthropometric profile of the participants.

**Table 1:** Characteristics of participants

Characteristic	Value	*n*	%
Gender	Boys	146	50.2
	Girls	145	49.8
Age	5 years	81	27.8
	6 years	70	24.1
	7 years	70	24.1
	8 years	70	24.1
	Mean ± SD	6.4 ± 1.14	
Weight (kg)	Mean ± SD	25.0 ± 4.98	
Height (cm)	Mean ± SD	121.8 ± 8.07	
BMI	Mean ± SD	16.69 ± 1.953	

Abbreviation: SD, standard deviation.

This study is based on a screening protocol to determine the foot posture of both feet using the FPI-6 [17]. Participants were allocated to one of the two-foot posture groups based on the screening protocol and qualified for the non-flatfeet normal group if static foot measurements were within one standard deviation of the mean of normative data for the FPI-6. Participants were assigned to the flexible flatfeet group if static foot measurements were greater than one standard deviation of the mean of normative data for the FPI-6.

The following functional tests were performed to assess the ability to correct deformities: (1) the great toe extension test (Jack’s test) and (2) the tip-toe standing test [13].

The study was conducted under the Declaration of Helsinki and approved by the local research ethics committee on April 08, 2019, under registration number BE-2-2. All parents and/or legal guardians of the participating children signed a written informed consent statement before these participants were brought into the study.

### 2.2 Clinical examination

The clinical examination was documented on a standard document sheet developed for routine use in orthopedic examination and was conducted in the same way for all patients. Parameter groups specifically for flexible flatfoot (FFF) diagnosis were evaluated in this study [3]: (1) passive ankle dorsiflexion and plantar flexion was measured with ruler-based goniometry. The patient was positioned supine, the knee extended (dorsiflexion knee 0°), and the ankle was redressed to the neutral position and the foot was prevented from supinating during dorsiflexion. The dorsiflexion was repeated with the knee flexed (dorsiflexion knee 90°). The dorsiflexion was performed looking for a tight Achilles tendon, thus differentiating between the equinovalgus and the planovalgus subtypes of FFF [18]. Less than 10° of dorsiflexion with the knee 0°, 90° suggests that the entire Achilles tendon is tightened. Less than 10° of dorsiflexion just with the knee 0° implies isolated gastrocnemius tightness [4]. (2) Torsional deformities: for tibial torsion deformity, the thigh-foot angle was calculated for both legs with the patient prone and the knees flexed to 90° and the ankle positioned in neutral [19]. A goniometer was placed along the long axis of the thigh and a line bisecting the calcaneus and ray of the second metatarsal. The angle between the second ray and the thigh axis was regarded as the thigh-foot angle. The negative value refers to the internal rotation of the tibia, whereas the positive value refers to the external rotation [20]. For the femoral torsion deformity, internal and external hip rotation was measured with the patient prone and the knees flexed to 90°. The hip midpoint of rotation was measured. Normal internal rotation of the hips limited to 50°. Raised internal rotation of the hip is considered to have an angle above 60° [3,21] and it is an indirect measurement for femoral anteversion in children. Moreover, it has the relationship between the flatfoot and femoral anteversion [21].

#### 2.2.1 Beighton

In this study, the Beighton hypermobility score was used to measure joint mobility. This scale consists of five items, with a total score ranging from 0 to 9 [22,23]. This scale is relatively insensitive and inappropriate for different ages, sex, and ethnic groups. A total score of >4 is used to define generalized hypermobility of joints in this study [22,24].

#### 2.2.2 Anthropometric measures

Height and weight were measured using a calibrated altimeter and digital scales with subjects wearing minimal clothing. The BMI was then calculated (weight (kg)/height (m^2^)).

#### 2.2.3 Foot posture assessments

The ND was used to measure the medial longitudinal arch according to Brody [5,10]. To evaluate the ND, the navicular height was measured by maintaining the subtalar joint in the neutral position under non-weight-bearing and weight-bearing conditions. ND test >9 mm represented a pronated foot type, 5–9 mm a neutral foot, and <5 mm a supinated foot [5]. This test has been reported to demonstrate moderate intra-tester reliability (intra-class correlation coefficient 0.61–0.79) and fair inter-rater reliability (0.57) [5,22,25].

The FPI-6 was used to evaluate the weight-bearing foot posture in the standing position [11]. Each criterion of the FPI-6 is scored on a 5-point scale (ranging from −2 to +2), and the scores are summed to provide a total score (ranging from −12 to +12) for the determination of foot posture [1] [22,26]. The FPI-6 is commonly used in research and clinical practice. It can be highly pronated (+10 to +12), pronated (+6 to +9), normal (0 to +5), supinated (−1 to −4), and highly supinated (−5 to −12). The index has been reported to demonstrate good reliability in adults and children [27]. Inter-observer reliability for the FPI-6 in the pediatric population is reflected in the consistent weighted Kappa value obtained (*K*_w_ = 0.86) by Morrison and Ferrari in a sample of children aged 5–16 years [27,28]. For every child in this study, the FPI-6 measurements were obtained for both feet by one experienced podiatrist.

RCSP was assessed based on the method described by Root [29]. Briefly, participants were asked to lie face-down on a bed parallel to the ground with their feet over the edge of the bed. An investigator examined their feet manually and put three dots on the upper, middle, and lower parts of the calcaneus to draw a bisection line regardless of fat around the calcaneus. RCSP was measured when individuals were standing with their feet fist-width apart. The angle between the bisector of the calcaneus and the perpendicular line to the ground was measured. Flatfoot was defined when either of the feet had greater than 4° valgus of RCSP angle [1,30].

#### 2.2.4 Footprint-based examination

Each patient was asked to put both feet on two Harris and Beath footprint mats while sitting on a chair [31]. The child then stood up to a standing position with even weight on both feet and returned to the sitting position to complete the footprint recording. Footprint data were rejected when apparent overshoot or imbalance had occurred on standing up or significant foot movement had occurred during recording. The footprint data from both feet were used [32]. Then the CSI and SI were used to measure the flatness of the footprint. The foot arch index measurements had previously shown excellent inter-rater reliability (SI 0.95; CSI 0.98) and test–retest reliability (SI 0.96; CSI 0.97) [32].

The CSI is the ratio between the smallest length of mid-foot and the largest length of the metatarsal head regions. Five categories are described for the medial longitudinal arch classification according to CSI: 0%: foot with elevated arch; 0.1–29.9%: foot with a morphological normal arch; 30–39.9%: intermediate foot; 40–44.9%: foot with a lowered arch; and 45% or higher flatfoot [14].

The SI is the ratio between the smallest length of the mid-foot and the largest length of the heel. Values between 0.44 and 0.89 were considered as normal values [14].

#### 2.2.5 Radiological measurements

Two bilateral radiographs consisting of lateral and anteroposterior views were obtained with the children in the bipedal standing position under weight-bearing conditions. On lateral weight‐bearing radiography, three angles were obtained: the talo-first metatarsal angle (TMA), talo‐calcaneal angle (TCA), and CP angle [33]. Flatfoot was defined as one of the following abnormal radiological findings: TCA > 45°, TMA > 4°, or CP < 20° [17,34]. The lateral X-ray measurements have a wide variation inter-rater reliability (TCA 0.568; TMA 0.46; CP 0.95) and intra-rater reliability (TCA 0.66; TMA 0.68; CP 0.96) in the assessment of pediatric flatfoot deformity [35].

The main descriptive characteristics of analyzed indicators under study are shown in Table 2.

**Table 2:** Descriptive characteristics of analyzed indicators

Indicators	Mean	SD	Normality		Percentiles		
			Skewness	Kurtosis	25th	50th	75th
Foot posture index (FPI-6)	5.49	3.948	−0.06	−1.68	2.0	7.0	9.0
Staheli arch index (SI)	1.05	0.37	0.13	−1.11	0.7	1.2	1.3
Chippaux–Smirak index (CSI)	60.27	21.31	−0.05	−1.70	38.2	69.5	79.2
Navicular drop (ND) test	10.91	5.410	−0.08	−1.66	5.0	12.0	16.0
Resting calcaneal stance position (RCSP)	5.62	2.969	−0.16	−0.89	3.0	6.0	8.0
Beighton score (hypermobility)	4.06	1.669	0.65	0.22	3.0	4.0	5.0
Tibial torsion (thigh-foot angle)	4.24	8.317	0.17	0.35	−2.0	5.0	9.0
Foot dorsiflexion (knee 90°)	26.27	3.384	0.14	−0.07	25.0	25.0	30.0
Foot dorsiflexion (knee 0°)	17.35	2.778	−0.17	−0.13	15.0	17.5	20.0
Foot plantar flexion	38.86	3.967	−0.17	−0.10	36.0	40.0	40.0
Internal hip rotation	54.53	11.521	0.42	0.26	45.0	55.0	60.0
External hip rotation	47.40	9.099	1.02	0.57	42.0	45.0	52.0
Calcaneal pitch (CP)	16.46	6.211	0.11	−1.20	10.0	17.0	22.0
Lateral talo-first metatarsal angle (TMA)	12.51	9.582	0.65	−0.93	4.0	9.0	21.0
Talocalcaneal angle (TCA)	44.20	10.900	−0.11	−1.15	34.0	45.5	53.0

Abbreviation: SD, standard deviation.

The mean of the FPI-6 was 5.5 pts, and the mean of the Beighton score was 4.1 pts.

### 2.3 Data analysis

Statistical analysis was performed with “IBM SPSS Statistics for Windows” (IBM Corp. Released 2017. IBM SPSS Statistics for Windows, Version 25.0. Armonk, NY: IBM Corp.). The descriptive analysis included means ± standard deviations (SD), the percentiles for continuous data as well as check for normality using skewness and kurtosis indicators. The categorical variables were described in absolute numbers (*n*) and percentages.

The comparison of means was conducted using Student’s *t*-test for independent samples concerning Levene’s test for equality of variances. The strength of associations between particular indicators and FPI was assessed using three ways: (1) correlation coefficient, (2) effect size, and (3) linear regression. The bivariate associations with FPI were assessed using Pearson’s correlation coefficient. The effect sizes were calculated using Cohen’s *d* coefficient. The multivariate analysis included linear regression modeling. The three scenarios to define the strongest associates of FPI were chosen to see how consistent the findings are and if the strongest associates are robust independently from analytical scenarios.

The level of statistical significance was set at *p* < 0.05.

## 3 Results

The study included 301 children with normal and flexible flatfeet. By comparing these two groups, majority of indicators differed significantly – footprint-based parameters (SI, CSI, *p* < 0.001), foot anthropometric parameters (ND, RCSP, *p* < 0.001), radiological foot parameters (CP, TMA, TCA, *p* < 0.001), and hypermobility parameter (Beighton score, *p* < 0.001). The majority of associated indicators showed highly significant differences between study groups (Table 3).

**Table 3:** Comparison of study indicators in children with flat and non-flatfeet

Indicators	Non flatfeet		Flatfeet		Difference	
	Mean	SD	Mean	SD	*t*	*p*
Staheli arch index (SI)	0.68	0.12	1.35	0.20	−49.35	<0.001
Chippaux–Smirak index (CSI)	37.71	3.84	78.75	7.38	−86.22	<0.001
Navicular drop (ND)	5.23	1.31	15.56	1.93	−76.71	<0.001
Beighton score (hypermobility)	3.69	1.70	4.37	1.58	−5.00	<0.001
Tibial torsion (thigh-foot angle)	4.69	7.97	3.87	8.59	1.19	0.234
Foot dorsiflexion (knee 90°)	26.40	3.50	26.17	3.29	0.81	0.417
Foot dorsiflexion (knee 0°)	17.51	2.83	17.22	2.73	1.26	0.207
Foot plantar flexion	38.50	4.11	39.16	3.82	−1.99	0.047
Internal hip rotation	53.00	11.10	55.79	11.72	−2.92	0.004
External hip rotation	46.80	8.83	47.89	9.30	−1.44	0.151
Resting calcaneal stance position (RCSP)	2.90	1.72	7.84	1.63	−35.54	<0.001
Calcaneal pitch (CP)	22.34	3.00	11.65	3.37	39.95	<0.001
Lateral talo-first metatarsal angle (TMA)	4.64	2.73	18.95	8.28	−29.04	<0.001
Talocalcaneal angle (TCA)	34.54	6.73	52.11	6.31	−32.43	<0.001

Abbreviation: SD, standard deviation; *t*, Student’s *t*-test.

Statistical analysis showed a significant FPI score correlation with other parameters (SI, CSI, RCSP, ND, CP, TMA, and TCA showed strong and moderate correlations, *p* < 0.001) based on Pearson’s coefficient (*r*). Because of similarity, effect sizes (*d*) among indicators were counted and the largest were CSI (*d* = 6.98), SI (*d* = 4.02), ND (*d* = 6.27), and CP (*d* = 3.35). Overall, the correlation and effect size calculations revealed very consistent patterns (Table 4).

**Table 4:** Study indicators and FPI: correlations and effect sizes

Indicators	*r*	*p*	*d*
Staheli arch index (SI)	0.862	<0.001	4.02
Chippaux–Smirak index (CSI)	0.919	<0.001	6.98
Navicular drop (ND)	0.913	<0.001	6.27
Beighton score (hypermobility)	0.173	<0.001	0.42
Tibial torsion (thigh-foot angle)	−0.052	0.211	0.10
Foot dorsiflexion (knee 90°)	−0.033	0.429	0.07
Foot dorsiflexion (knee 0°)	−0.053	0.203	0.10
Foot plantar flexion	0.092	0.027	0.17
Internal hip rotation	0.123	0.003	0.24
External hip rotation	0.090	0.029	0.12
Resting calcaneal stance position (RCSP)	0.781	<0.001	2.95
Calcaneal pitch (CP)	0.826	<0.001	3.35
Lateral talo-first metatarsal angle (TMA)	0.720	<0.001	2.32
Talocalcaneal angle (TCA)	0.774	<0.001	2.69

Abbreviation: *r*: Pearson’s correlation coefficient, *d*: Cohen coefficient for the effect sizes.

To eliminate possible interaction effects between the analyzed indicators, we conducted a multivariate analysis. The linear regression revealed the same pattern – that the strongest associates are footprint-based parameters CSI (*β* = 0.34) and ND (*β* = 0.28). Other strong correlations are SI and CP, with all other indicators having relatively small relationships with the FPI (Table 5).

**Table 5:** Comparison of study indicators as potential predictors of FPI: linear regression analysis

Indicator	*B*	*β*	*p*	VIF
Staheli arch index (SI)	1.65	0.16	<0.001	4.82
Chippaux–Smirak index (CSI)	0.06	0.34	<0.001	8.22
ND (ND)	0.20	0.28	<0.001	8.53
Beighton score (hypermobility)	−0.04	−0.02	0.253	1.09
Tibial torsion (thigh-foot angle)	−0.01	−0.02	0.114	1.07
Foot dorsiflexion (knee 90°)	0.01	0.01	0.754	2.36
Foot dorsiflexion (knee 0°)	−0.03	−0.02	0.393	2.39
Foot plantar flexion	0.01	0.01	0.603	1.19
Internal hip rotation	0.00	0.00	0.887	1.12
External hip rotation	0.01	0.02	0.108	1.10
Resting calcaneal stance position (RCSP)	0.06	0.04	0.067	3.16
Calcaneal pitch (CP)	0.07	0.12	<0.001	3.58
Lateral talo-first metatarsal angle (TMA)	0.01	0.03	0.112	2.36
Talocalcaneal angle (TCA)	0.02	0.05	0.035	2.92

Abbreviation: VIF, the variance inflation factor.

## 4 Discussion

The FFF topic, especially asymptomatic form, is under very tense discussion widely by doctors, representatives of various fields of science, scientists, and parents of anxious children. However, this debate is still stuck at the lowest level of the evidentiary pyramid in evidence-based medicine and to treat or not to treat FFF is in the experienced physician’s hands – opinion [2]. The strength of the present study is that the topic of the study is of importance to practitioners given the high number of (clinical, anthropometrical, and radiological) presentations to pediatric orthopedic services that are related to lower limb conditions. The foot only constitutes 14% of all the musculoskeletal consultations and parental concerns regarding foot development are high on that list [36]. Parents who are often worried about the child’s feet posture bring the child to an orthopedic. These children are usually with “abnormal” rotational deformities of the lower extreme [6,21,37]. Zafiropoulos et al. [21] showed that children, aged between 3 and 6 years with a raised internal rotation of the hip (≥60°) and as an indirect measurement of femoral anteversion, have a relationship between flatfoot and femoral anteversion (*F* = 168.1, *p* < 0.001, *r* = 0.53). They concluded that it is necessary to investigate further with ages above 6 years. In our study, the FPI-6 tool was used to include the patients instead of footprint measurement contact index II. The internal rotation of the hip was 55.79° ± 11.72 in the flatfoot group and 53.00° ± 11.10 in the non-flatfoot group. This is statistically nonsignificant and we did not notice any statistically significant correlations with the FPI.

Some studies [38,1] assessed the correlation between FPI-6 and RCSP angle in elementary school students (8–13 years) and adolescents (11–16 years), and declared moderate correlation. In the current study, we also found a moderate correlation to FPI-6 (flatfeet *r* = 0.781 and *r* = 0.714, respectively, *p* < 0.001). However, no correlation was observed between the first three measures after comparing the effect sizes. This raises doubts, because FPI-6 that is proven with validity and reliability is widely used by clinicians. On the contrary, RCSP angle is used by orthotic specialists. Furthermore, Cho, Park, and Nam found no statistically significant association of BMI with FPI-6. Martinez-Nova et al. [39] have also shown the FPI-6 minimal relationship with weight, height, and BMI. Because of this fact, we have not searched the relationships between these factors. Yet, several reports have shown that obesity and overweight children typically display flatter feet relative to their leaner counterparts; however, the cause of their flatter feet is unknown [38,40,41,42].

In the current study, we demonstrated that the ND and footprint indexes – CSI and SI –  had the strongest correlations with FPI-6 in flatfoot children and a moderate relationship in non-flatfoot children. This finding is consistent with other recent findings by our group. It agrees with other studies [10,38], where the correlation between the ND and the FPI-6 was excellent (*P* < 001; *r* = 0.818), including that between the footprint parameters and the FPI-6 was good as well (*P* < 0.001; *r*_s_ = |0.663–0.703|). Contrary to findings in our study and based on the data of the study by Langley, Cramp, and Morrison, [5], it was recently reported that an ND is not an acceptable measure for characterizing the foot. Interestingly, ND was the least consistent measure for classifying the foot (*K*_w_ = 4). As opposed to our study, the ND test was the second choice indicator as a potential predictor of FPI-6. It might be explained by age, where the adult population was studied (29 ± 6 years). However, the vast majority of studies in literature have been conducted with adults. As children’s feet are changing over time (cessation of time is unknown [2]), comparisons made with adults can mask other related factors. Further research is needed.

On the contrary, most of the studies have highlighted the frequent use of FPI-6 as a sensitive, specific, and predictive tool in the evaluation of pediatric FFF, which is very important not only for researchers but also for clinicians [43,44]. However, in the pediatric population, this excellent diagnostic tool has its limitations in terms of its sensitivity at different ages of the child. Limitations occur when the natural history of the pediatric FFF, as a morphology that usually reduces with age, is not taken into account [2]. The FPI-6 score would be predicted to change with each year of childhood [2,39]. Martínez-Nova et al., in the prospective 3-year study [39], demonstrated a pronated and highly pronated FPI-6 category where the pediatric foot had converted to a neutral FPI-6 foot type as age increased. They declared that pronated foot posture can be expected in children aged less than 9–10 years and there can be a spontaneous reduction without any treatment. Other studies also confirm this [36,45]. What is more, in clinical practice, is that this fact should be a strong signal to the pediatric orthopedist, because it highlights the need for caution when interpreting results based on the FPI-6 diagnostic tool in the pediatric population. A future follow-up of patients as they grow older is necessary. Despite this important observation, the majority of existing studies have generally included the larger age gap of children such as 6–18, 3–15, and 3–17 years old [2,44]. The results do not emphasize at what age gap the pediatric foot posture changes during development. Therefore, much of the existing literature has limited external validity for practitioners working with children with FFF. Our research addresses two of these discussed concerns by including non-flatfeet and asymptomatic flatfeet participants of ages 5–8 where the majority of outcome measures are valid and reliable indicators of FFF or related to FFF.

Even though the high prevalence of flatfoot in children is related to both the anatomy of the foot variability [46] and the lack of diagnostic criteria and diagnostic test latitudes [47], the difference between symptomatic FFF and asymptomatic FFF remains an assessment of pain, fatigue in the child’s feet, and other subjective sensations [48]. Interestingly, differences in foot kinematics between symptomatic and asymptomatic FFF could not be found [3,49]. Bohm et al. [3] concluded the substudy that it is important to differentiate decompensated FFF, because those are more prone and require surgical interventions, even if they are not yet associated with pain. This means we need to go back to surgical planning and radiological evaluation pre- and post-surgery in asymptomatic FFF. However, studies on the correlation of FPI-6 with footprints, clinical evaluation, gait analysis, and radiographs are limited. In our study, lateral radiographic measurements of feet were included (TMA, TCA, and CP angles). Radiography is a highly reliable gold-standard measure for the assessment of the skeletal alignment of the foot in static weight bearing position [44]. All these angles had moderate-to-strong correlations with FPI-6 in our researched population. However, the linear regression analysis showed that CP has the highest potentiality to predict FPI-6. This conflicts the results of previous studies [33,44,50], where the TMA angle was chosen to represent the foot posture based on ease of measurement, good reliability, and the degree to which it reflects the static foot posture. Lee et al. [33] had found that RCSP significantly correlated with the TMA angle, but the CP angle was not significantly correlated with either TMA or TCA. However, they used RCSP in their study instead of FPI-6. Furthermore, they declared that TMA, the most predictable parameter obtained on simple X‐ray, was well correlated with TCA and RCSP. In general, clinical and radiologic measurements did not show significant correlations. This observation in our study found that the CP angle has the greatest potential to predict FPI-6. It allows future studies in the pediatric population to make a more targeted choice of a more valuable measured angle, as well as a more reliable choice for radiologists.

To the best of our knowledge, this paper also appears to be original in terms of participants and outcome measure approaches. The result is potentially valuable, particularly for practitioners, radiologists, rehabilitation professionals, orthotic shoe manufacturers, and those presenting with symptoms of FFF or asymptomatic FFF. Understanding the posture of the foot in developing children helps to detect any persisting deviations beyond a certain stage of development. It also provides a margin for timely intervention to avoid possible deformities and dysfunctions. Although there are several methods available, one of the main limitations with static measurements of foot posture is that generally only one clinical technique is used in each investigation. Because of the differences in sample characteristics and measurement procedures, it is difficult to compare the results of different studies and make an informed decision on the most appropriate technique. As the foot complex is almost developed in adolescence, there is a need for normative baseline data in this population, which can help to compare the deviations seen in children with impaired foot posture. These values can also be used to monitor the outcome of regular examinations for foot impairments. Finally, this research is one of the few in the area that is attempting to answer questions raised in the pediatric orthopedic community, which are listed in the systematic review paper by Uden et al. [2]. In multiple literature, FFF is a very targeted foot type and often “diagnosed” using one or more non-validated assessments [47]. This raised the first question regarding which of these foot measures, if any, should be used to assess the posture of the developing foot. The FPI-6 is a validated and accurate method for quantifying standing foot posture and determining pediatric FFF [27,44,51]. According to our study results from the footprint-based measures, the CSI had the strongest associations with FPI-6 as well as with the ND parameter. In addition, the CP angle had the strongest correlation from radiological foot posture characteristics in comparison to most usable parameters for diagnosing FFF. The second question was about the level of importance of these indicators and, if any, should be placed on the static posture of the developing foot, because of the notable absence of functional and clinical data in the literature. The use of foot anthropometric measurements is found to be a salient role in the analysis of the foot types using the ND, RCSP, and other parameters in the 5–8-year age group. Our study noted, next to FPI-6 which is very important method in daily practice [43], stands firmly and ND, CP, CSI, and SI in the pediatric 5–8 years population. However, this needs to be highlighted for caution when directly interpreting results.

This study had several limitations. First, this was a cross-sectional study. The enrolled sample and results cannot be extrapolated to the entire population. In addition, further longitudinal studies should be conducted to investigate between clinical, radiological, and gait analysis assessments. Second, all measurements were performed by the same practitioner and recorded as one measurement. Intra-rater reliability was not confirmed in FPI and other measurements. The high reliability of FPI-6 is described in the literature [52]. Third, we did not include children with symptomatic FFF. In the literature review, we did not succeed to find any articles evaluating the totality of visual, anthropometric, clinical, radiological, and gait analyses indicators in the study. Nothing was found by comparing the characteristics of normal feet, asymptomatic FFF, and symptomatic FFF, especially studies on larger sample size in larger and varied geographical regions. Finally, all measurements and examinations of the patient were performed in 1 day. By assessing the scope of the data and the age of the children (aged 5–8 years), it could have affected error even if there were breaks between measurements.

## 5 Conclusions

A positive correlation was observed between FPI-6 and ND test, CSI in 5–8-year-old children. In addition, all three prominent foot posture indicators (FPI-6, ND, and CSI) might be used as primary or preferred tools in clinical practice determining the shape of the foot. This is the first research investigation that shows a correlation between FPI-6 and other clinical, anthropometrical, and radiological parameters together in children at 5–8 years. There is potential for future research in this field with more longitudinal designs and across different samples and races to see if these findings are consistent or specific to certain subgroups.

## Abbreviations

BMI: body mass index
CP: calcaneal pitch angle
CSI: Chippaux–Smirak index
FFF: flexible flatfoot
FPI-6: foot posture index six
ND: navicular drop
RCSP: resting calcaneal stance position
SI: Staheli arch index
TCA: talocalcaneal angle
TMA: lateral first metatarsal angle

## Appendix

**Table A1:** Study parameters of the investigations into paediatric foot posture and foot posture index six (FPI-6), show that FPI-6 and footprint measures have dominated foot posture assessment

Study: author, country	Year of publication	Design	Participans (n)	Age (years)	Method of foot posture assessment	Highlights of the study
Fatma A Hegazy et al., United Arab Emirates, Egypt	2020	A cross-sectional	612	6–18	FPI-6Foot X-ray: TMA	FPI-6 is valid and with moderate diagnostic accuracy for determining paediatric FFF
Yongjin Cho et al., Republic of Korea	2019	Retrospective comparative	208	8–13	FPI-6RCSP	Moderate correlation between FPI-6 and RCSP angle
Alfonso Martínez-Nova et al., Spain	2018	Prospective	1,032	5–118–14	FPI-6	Assessing FPI-6: children’s foot posture shifts toward neutral as age increases
Sriraghunath S et al., India	2018	A cross-sectional	391	11–16	FPI-6RCSPND	A large correlation both in adolescent girls and boys between FPI-6 and anthropometric measurements (ND, RCSP)
Fiona Hawke et al., Australia, New Zeland	2016	Cross-sectional	30	7–15	FPI-6	Children with a more pronated foot type exhibited greater lower limb and whole-body flexibility, but not greater ankle joint flexibility
Gill et al., USA	2016	Cross-sectional	254384	2–1718–80	Footprints: CSI, KIND	Arch height indices (CSI, KI) were correlated with navicular height (ND)
Isabel CN Sacco et al., Brazil, Germany	2015	Longitudinal for German populatio,Brazilian – Cross-sectional	94391	3–10	Footprints: CSI,SI	Revealed anthropometric differences in absolute forefoot and rearfoot widths of German and Brazilian children, but a similar longitudinal arch development
Angela M Evans, Australia	2011	Cross-sectional	140	7–10	FPI-6	Did not find a positive relationship between increased body weight and flatter foot posture
George Zafiropoulos et al., UK and Greece	2009	Prospective	621	3–6	Footprints: CI IIClinical: internal hip rotation	A highly significant correlation between flat foot and raised internal rotation of hip
Jung H. Lee et al., Korea	2009	Observational	13	6.6 ± 2.3	Clinical: femoral anteversion, femoral internal rotation, femoral external rotation, tibial torsion (thigh -foot angle)RCSPFoot X-ray: TMA, TCA, CP, metatarsal adductionCTGait	TMA was related to the degree of RCSP. Larger TCA contributed to decreased maximal external rotation and increased maximal internal rotation
